# Job vacancy at ECDC

**DOI:** 10.2807/1560-7917.ES.2022.27.46.221117v

**Published:** 2022-11-17

**Authors:** Eurosurveillance editorial team

**Affiliations:** 1 European Centre for Disease Prevention and Control (ECDC), Stockholm, Sweden

The European Centre for Disease Prevention and Control (ECDC) invites applications for the following position:

 

- **Data Protection Officer**
  The application deadline expires on 16 December 2022.

 

For more information, please visit the ECDC website: 

https://erecruitment.ecdc.europa.eu/?page=advertisement

